# End-of-shift surgical handover: mixed-methods, multicentre evaluation and recommendations for improvement

**DOI:** 10.1093/bjsopen/zrae023

**Published:** 2024-04-03

**Authors:** Jessica M Ryan, Anastasija Simiceva, Walter Eppich, Dara O Kavanagh, Deborah A McNamara

**Affiliations:** RCSI SIM Centre for Simulation Education and Research, 123 St. Stephen’s Green, RCSI, Co. Dublin, Ireland; RCSI StAR MD programme, St. Stephen’s Green, RCSI, Co. Dublin, Ireland; Department of Surgery, The Bon Secours Hospital, Glasnevin Hill, Glasnevin, Co. Dublin, Ireland; RCSI SIM Centre for Simulation Education and Research, 123 St. Stephen’s Green, RCSI, Co. Dublin, Ireland; RCSI SIM Centre for Simulation Education and Research, 123 St. Stephen’s Green, RCSI, Co. Dublin, Ireland; Department of Medical Education and Collaboratory Practice Centre, The University of Melbourne, Melbourne, Australia; RCSI Department of Surgical Affairs, 121 St. Stephen’s Green, RCSI, Co. Dublin, Ireland; Department of Surgery, Tallaght University Hospital, Tallaght, Co. Dublin, Ireland; Office of the President, RCSI, 123 St. Stephen’s Green, Co. Dublin, Ireland; National Clinical Programme in Surgery, RCSI, 2 Proud’s Lane, Co. Dublin, Ireland; Department of Surgery, Beaumont Hospital, Beaumont, Co. Dublin, Ireland

## Introduction

Handover is defined as ‘the exchange between health professionals of information about a patient’ and accompanies a transfer of responsibility for that patient^1^. Ineffective handover harms patients^2,3^, incurs significant monetary^4^ and opportunity costs^5^, and causes 40% of communication-related malpractice claims^6^. Surgical patients are vulnerable to such communication failures^7^.

Despite published handover guidelines^8,9^, implementation is poor and no ‘standard’ exists^4^. Previous studies have audited surgical handover^10,11^; however, few used qualitative methods to explore the impact of behaviours and context, and no interventional studies rigorously applied an implementation framework, so enablers and barriers remain poorly understood. A comprehensive mixed-methods evaluation of surgical handover is required to determine the causes of poor compliance with guidelines.

The aim of this study was to assess adherence to international guidelines during post-call general surgical handover, to identify barriers to guideline compliance and to develop recommendations for process improvements.

## Methods

A mixed-methods approach using triangulation of data sources^12^, through audit, focused rapid ethnographic evaluation (FREE)^13^, interviews, focus groups^14^ and a survey was utilized to assess post-call surgical handover and develop recommendations for improvements (*Supplementary materials, Text*  *S1*).

### Setting and participants

This study was conducted in two Irish university teaching hospital surgical departments. Both sites are tertiary referral centres with 72 general surgical non-consultant hospital doctors (NCHDs) participating in handovers. Prospective study approval was received at both sites (3445, CA2023/040).

### Study design

During phase I (January–April 2023), handovers were observed and assessed using a bespoke data collection tool (*Supplementary materials, Appendix S2*) and real-time coding method (*Supplementary materials,*  *Table S3*), recording audit and ethnographic (FREE) data. Guidelines were used to assess handover structure and verbal content (*Supplementary materials,*  *Table S4*). FREE was used to assess behavioural and contextual factors through observations and field interviews, refined based on observational findings of the handover process. During phase II (June–July 2023), additional data were gathered through interviews, focus groups and a staff survey. Phase I findings were presented to participating NCHDs to elicit feedback and evaluate barriers and facilitators of handover. A cross-sectional, open-ended, staff survey asked: ‘What would you like to change about handover in your hospital? Do you have any suggestions for improvement?’.

### Data analysis

Pooled data from interviews, focus groups and survey responses were analysed using inductive thematic analysis^15^. Quantitative data were analysed using Stata (17.0©2021, StataCorp, TX). Descriptive data are presented as absolute values and percentages, with continuous data presented as mean (standard deviation) or median (range). Comparative analyses of quantitative data were performed using chi-squared tests (two-tailed, significance level *P* < 0.05).

## Results

In total, 208 individual patient handovers from 26 handover meetings were assessed, encompassing 283 min of observations over 87 separate days; 23 interviews were carried out, and 24 survey responses were provided (43.6% response rate). Three separate focus groups including eight senior registrars were completed. Qualitative data were obtained from 73.3% (*n* = 11) of senior registrars (that is handover leaders).

### Handover observations

Mean compliance scores for handover setup, structure and verbal content were 53.3%, 30.3% and 33.1% respectively (*Table 1*).

**Table 1.** Compliance with handover guidelines for handover setup, content, and structure

Recommendation category	Compliance
**Setup – Timing (n=26 meetings)**	
Handover should be at a fixed time *(occurring within a 30-minute window)*	61.5 (16)
Punctuality is a key requirement *(any delay >1 minute)*	23.1 (6)
Bleep-free/protected time should be enforced	0 (0)
There should be a handover every morning *(n=27 mornings)*	96.3 (26)
Handover is expected to take 30 minutes or less	100 (26)
**Setup – Attendance (n=26 meetings)**	
Handovers should be multidisciplinary	0 (0)
All necessary grades of staff should attend	0 (0)
There should be involvement of the nurse clinical coordinator/bed manager	0 (0)
With daily senior clinician involvement *(consultant attendance)*	11.5 (3)
**Setup – Leadership (n=26 meetings)**	
There should be clear leadership, supervised by most senior clinician present	100 (26)
**Setup – Location (n=26 meetings)**	
It should be conducted verbally and face-to-face where possible	100 (26)
It should take place in a dedicated space *(not in use by others)*	3.8 (1)
Large enough to allow everyone to attend	100 (26)
Free from distractions *(extraneous noise)*	65.4 (17)
Location should maintain patient confidentiality	100 (26)
**Setup – Available patient information resources & handover tools (n=26 meetings)**	
Working computers	100 (26)
Clinical information/electronic records	100 (26)
Telephones	100 (26)
Relevant documentation *(i.e., an available electronic handover)*	57.7 (15)
Patient census sorted by location which identifies urgency of review	0 (0)
A handover checklist	0 (0)
**Handover structure (n=25 meetings)**	
Leader to begin with a short introductory briefing to facilitate situational awareness	12 (3)
Team members should be alerted to clinically unstable patients at the outset	4 (1)
There should only be one speaker at a time	92 (23)
Two-way communication process – There should be questions/input from the team	68 (17)
Tasks should be prioritised	24 (6)
Operational issues should be discussed	12 (3)
The leader should promote read back of patient information, to verify receival	0 (0)
**Verbal handover content (n=208 patient presentations)**	
A handover method should be used *(e.g., ISBAR, I-PASS)*	0 (0)
Info given should be succinct/relevant *(n=6 items or less, relevancy not assessed)*	79.8 (166)
Patient condition/illness severity should be clarified at the outset of each presentation	3.8 (8)
Current diagnosis *(diagnosis or differential diagnosis provided)*	8.6 (18)
Relevant background (*any background given)*	46.6 (97)
Results of significant or pending investigations *(any details of assessment)*	55.7 (116)
Anticipated issues/contingency plans	11.5 (24)
Management plans	84.6 (176)
Outstanding tasks assigned	7.2 (15)

Values are % (n). I-PASS, illness severity, patient summary, action list, situation awareness and contingency planning, synthesis by receiver^16^; ISBAR, identity, situation, background, assessment, recommentation^17^.

#### Handover setup

A handover meeting usually occurred, but neither site implemented protected handover time. Punctuality was poor, with delays caused by late staff and issues with documentation. No meetings were attended by all required grades of staff and only 11.5% (*n* = 3) were attended by a consultant. During handovers, registrars sometimes excluded interns, who struggled to hear the handover or did not participate at all. On one occasion, the on-call team were operating until just before handover, meaning several patients were awaiting review and the electronic handover (e-handover) was incomplete. Hospital A had significantly fewer available e-handovers (35.7%, *n* = 5 *versus* 83.3%, *n* = 10; *x^2^* = 6, *P* = 0.014; *Supplementary materials, Table S5*).

#### Handover structure and content

Introductory briefings, task prioritizations and summary statements were seldom provided, the sickest patients were rarely prioritized for discussion, and no readbacks took place (*Table 1*). In total, 208 patient handovers were coded for 16 categories of information (908 codes). The content and order of handover was highly variable. Almost no presentations began with illness severity and only 48.5% ended with a recommended plan. Contingency plans and action assignments were lacking (*Supplementary materials, Fig. S6*). A median of 0.5 (0–2) interruptions occurred per handover, mostly caused by consultants.

### Qualitative analysis

Thematic analysis of coded data enabled identification of two major themes (*Supplementary materials,*  *Table S7*).

#### Significant invisible work surrounds the handover process

Interns needed up to 30 min to prepare for handover. On-call senior house officers completed and circulated the e-handover, attended pre-handover ward rounds, managed last minute referrals and covered multiple surgical subspecialties.

#### Factors influencing effective handover practice

Documentation and information technology (IT) issues were common with inaccurate or unavailable documentation, caused by time constraints and limited intern access to e-handovers. Staff reported difficulty accessing computers and printers, unavailable login details and unfamiliarity with systems. Senior registrars at Hospital B attributed good e-handover completion to departmental culture and consultant oversight. Staff were eager for electronic patient records and increased automation.

Time and handover location were variable, interfering with IT access, causing increased distractions and patient confidentiality concerns. Standardized time and location provided predictability for staff. They were also eager for implementation of protected handover time to facilitate pre-handover activities. Consultant and junior staff engagement was reported to be poor at times. A strong handover culture and consultant oversight were considered important enabling factors.

### Recommendations for handover improvement

**Fig. 1:**
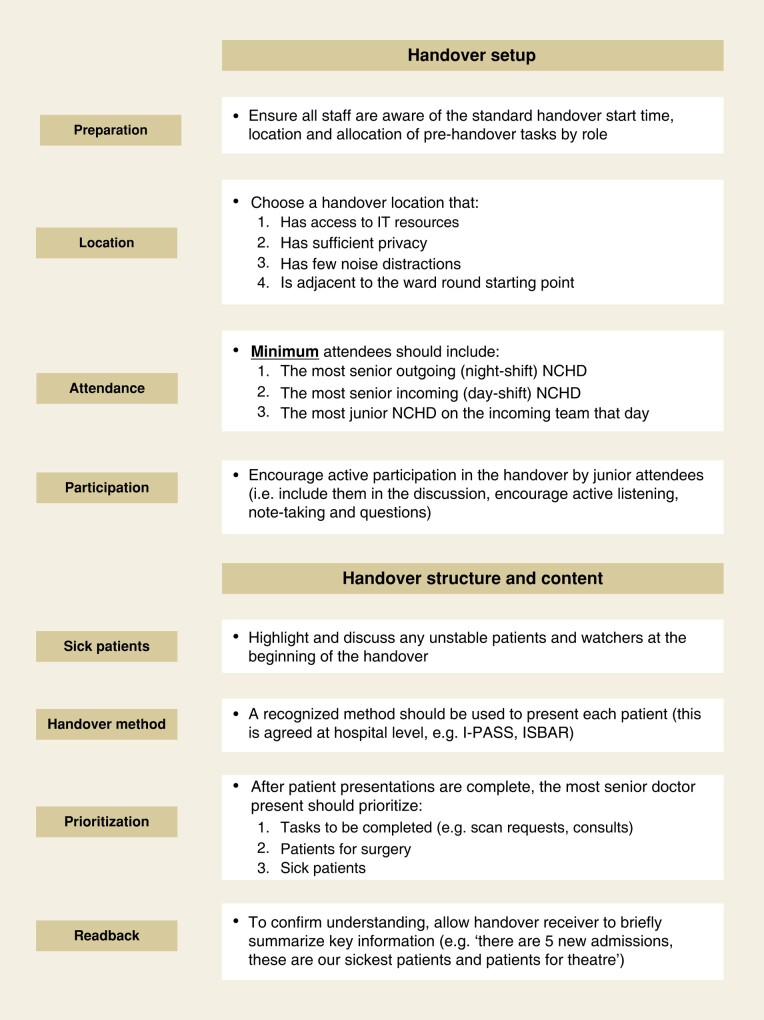
Recommendations for handover improvement^16,17^ IT, information technology; NCHD, non-consultant hospital doctor; I-PASS, illness severity, patient summary, action list, situation awareness and contingency planning, synthesis by receiver; ISBAR, identity, situation, background, assessment, recommentation.

Based on the study findings and available guidelines, recommendations to assist surgical handover leaders to optimize the process and reduce variability in performance are reported (*Fig. 1*).

## Discussion

This multicentre, mixed-methods study identified aspects of surgical handover that do not align with best practice guidelines and explored barriers and enablers of improvement. While face-to-face handovers with clear leadership usually occurred, poor compliance with best practice was observed (*Table 1*). Handovers lacked structure, omitted introductory briefings and prioritization, lacked emphasis on unwell patients and did not summarize findings. Much invisible work by junior staff is required to prepare for handover but is adversely affected by time constraints, challenges with documentation, variability in handover time and venue, and unexpected clinical activities. Key changes recommended by staff included protected handover time, standardization of time and location, and senior oversight, all of which are recommended by handover guidelines. Existing guidelines do not acknowledge that NCHDs are key stakeholders in this process but implementation of many recommendations is beyond their control. Handover leaders require support in implementing best practice guidance in resource-limited situations. To address this, new recommendations are reported.

Several studies have used audit to evaluate handover^10,11^; however, quantitative assessments of complex team-based processes yield limited information, and provide only superficial understanding of barriers and facilitators. No previous interventional studies of daily surgical handover have applied an implementation framework, an approach that requires in-depth understanding of local context^18^. The replicable methodology described in this study will enable other clinicians to use this approach, even in personnel- and resource-constrained settings. The clear patient safety risk of poor handover practices^2,3^ means that hospital self-assessments are required to determine guideline compliance and identify areas for improvement. The methodology described provides a rapid, cheap and straightforward method to achieve this.

This study identified important deficiencies and barriers in the surgical handover process and provides clear recommendations to support handover leaders. The need for rigorous assessment of handover practices cannot be overstated, given its important role in safe surgical care and healthcare efficiency. The combination of methodologies used in this study offers a fresh perspective and a robust framework for future handover assessment studies.

## Supplementary Material

zrae023_Supplementary_Data

## Data Availability

The authors agree to make the data, analytic methods and study materials available to other researchers. These can be obtained by contacting the corresponding author using the details provided. Additional data has not been published in a public repository. The authors did not preregister this study in an independent, institutional registry as it was approved by the quality and safety department of the hospital and did not require research ethics approval.
