# Supplementary material for: End-of-shift surgical handover: mixed-methods, multicentre evaluation and recommendations for improvement
Source: BJS Open. 2024 Apr 3;8(2):zrae023. doi: 10.1093/bjsopen/zrae023 (PMC10989866; doi:10.1093/bjsopen/zrae023)
Supplement: zrae023_Supplementary_Data [file zrae023_supplementary_data.zip › GRAMMS checklist.docx]

Good Reporting of A Mixed Methods Study (GRAMMS) checklist

Title: **A mixed-methods, multi-centre evaluation of end-of-shift surgical handover using rapid ethnography and audit: Recommendations for improvement**

| Guideline | Section: page |
| --- | --- |
| Describe the justification for using a mixed methods approach to the research question | Introduction: Page 4 |
| Describe the design in terms of the purpose, priority and sequence of methods | Methods: Page 5-6, supplementary text S1 |
| Describe each method in terms of sampling, data collection and analysis | Methods: Page 5-6, supplementary text S1 |
| Describe where integration has occurred, how it has occurred and who has participated in it | Methods: Page 5-6, supplementary text S1 |
| Describe any limitation of one method associated with the presence of the other method | Discussion: Page 10, supplementary text S1 |
| Describe any insights gained from mixing or integrating methods | Discussion: Page 10-11 |

*Ref: O'Cathain A, Murphy E, Nicholl J. The quality of mixed methods studies in health services research. J Health Serv Res Policy. 2008;13: 92-98*
