# Supplementary material for: End-of-shift surgical handover: mixed-methods, multicentre evaluation and recommendations for improvement
Source: BJS Open. 2024 Apr 3;8(2):zrae023. doi: 10.1093/bjsopen/zrae023 (PMC10989866; doi:10.1093/bjsopen/zrae023)
Supplement: zrae023_Supplementary_Data [file zrae023_supplementary_data.zip › Supplemental material.docx]

**A mixed-methods, multi-centre evaluation of end-of-shift surgical handover: Recommendations for improvement**

Jessica M Ryan^1, 2, 3^, Anastasija Simiceva^1^, Professor Walter Eppich^1, 4^, Mr. Dara O Kavanagh^5, 6^, Professor Deborah A McNamara^7, 8, 9^

**AUTHOR INSTITUTIONS**

^1^ RCSI SIM Centre for Simulation Education and Research, 123 St. Stephen’s Green, Co. Dublin

^2^ RCSI StAR MD programme, St. Stephen’s Green, Co. Dublin

^3^ The Bon Secours Hospital, Glasnevin Hill, Glasnevin, Co. Dublin

^4^ Faculty of Medicine, Dentistry, and Health Sciences, University of Melbourne, Australia

^5^ RCSI Department of Surgical Affairs, 121 St. Stephen’s Green, Co. Dublin

^6^ Department of Surgery, Tallaght University Hospital, Tallaght, Co. Dublin

^7^ Office of the President, RCSI, 123 St. Stephen’s Green, Co. Dublin

^8^ National Clinical Programme in Surgery, RCSI, 2 Proud’s Lane, Co. Dublin

^9^ Department of Surgery, Beaumont Hospital, Beaumont, Co. Dublin

**CORRESPONDING AUTHOR**

Name: Jessica M Ryan

Address: RCSI SIM Centre for Simulation Education and Research, 123 St. Stephen’s Green, Co. Dublin, Ireland

Email: [jessicaryan@rcsi.com](mailto:jessicaryan@rcsi.com) ORCID: <https://orcid.org/0000-0001-6161-9630>

Twitter: @jessmryan

**Supplementary Materials - Index**

| **Supplementary Appendixes** |  |
| --- | --- |
| Supplemental methods and discussion (Text S1)  Handover observation tool (Appendix S2) | *pag. 3-10*  *pag. 11-12* |
|  |  |
| **Supplementary Figures and Tables** |  |
| Handover observation codes (Table S3)  Summary of guideline recommendations used to guide observations (Table S4) | *pag. 13*  *pag. 14-15* |
| Observations relating to the handover setup assessed using audit and ethnography (Table S5)  Content and order of observed handovers (Figure S6)  Qualitative findings (Table S7) | *pag. 16*  *pag. 17*  *pag. 18-20* |
| **References** | *pag. 21-24* |
|  |  |

**Supplementary Appendixes**

**Text S1**

**METHODS**

A mixed-methods approach using triangulation of data sources^1^ was utilised to assess the post-call surgical handover. Findings were reported using the Strengthening the Reporting of Observational studies in Epidemiology (STROBE)^2^ and Good Reporting of A Mixed Methods Study (GRAMMS)^3^ guidelines. During phase I, carried out between January and April 2023, a set of handovers were observed and assessed using a data collection tool for recording both quantitative (audit) and qualitative (rapid ethnographic) data. During phase II, between June and July, additional data were gathered through interviews, focus groups, and a staff survey. Quantitative and qualitative findings formed the foundation of recommendations for process improvements.

**Setting and population**

This study was conducted in surgical departments of two university teaching hospitals in Dublin, Ireland. Both sites are busy tertiary referral centres, with 500 and 820 beds, and catchment populations of 644,000 and 290,000 people, respectively. A total of 72 general surgical non-consultant hospital doctors (NCHDs) participated in the post-call handovers across both hospitals sites. Prospective approval for this study was received from the quality departments at both sites (3445, CA2023/040).

**Study design**

*Audit of handover*

During phase I, the handover process was audited for compliance with national and international guidelines (Table S1)^4-9^ through direct observations of handover meetings. The study team selected guideline recommendations for inclusion if handover leaders could influence them directly (e.g., handover location), or if measurement was simple (e.g., implementation of a bleep-free period). Written handovers were not assessed. The I-PASS handover method^10^ was used as a standard to assess the order of content included in the handover, as guidelines do not stipulate this (Table S1). Staff required to be present at handover were defined as surgical doctors closely involved in the care of patients reviewed during the on-call period, or those involved in caring for these patients during the upcoming shift. ‘Outgoing’ staff were those finishing their on-call or overnight shift (post-call staff), and ‘incoming’ staff were those about to begin the day-shift. The ‘handover leader’ was defined as the most senior person present, in charge of managing the handover discussion.^8^

*Focused Rapid Ethnographic Evaluation*

Ethnography was used during phase I of this study, through handover meeting observations and field interviews. The definition of ethnography is elusive^11^, but for the purposes of this study, related to immersion of the observer in the environment of the handover meetings, with assessment of the behaviours and contexts which were perceived to influence the handover process. Specifically, Focused Rapid Ethnographic Evaluation (FREE) was used to minimise disruption to staff in the clinical environment. FREE is similar to other methods of rapid ethnography^12^; however, it utilises field notes instead of digital recordings^13^, protecting patient and staff confidentiality. Audit and ethnography were used simultaneously to assess the same set of handover meetings. International guidelines provided a framework for the assessment (see *development of the observational tool*), and ethnography was used to interpret and elaborate upon these findings. These data have been reported concurrently (see *Handover observations*). Qualitative data obtained from field interviews were pooled with other sources for analysis.

**Data collection**

*Development of the observational tool*

The tool used in phase I was developed through a two-week pilot period during which unstructured observations were carried out to assess study feasibility and determine which variables could be efficiently captured using fieldnotes. After four observations, the study team created an early version of the tool, informed by guideline recommendations (Table S1), and further refined the tool through an iterative process over a two-week period (Appendix S2). This tool was used to record both audit and ethnographic data.

*Handover observations*

Overt, structured, non-participant observations of surgical handovers were conducted by JR - a surgical trainee familiar with both hospital sites but currently employed in a full-time research role. Post-call, weekday, general surgical handovers were assessed. During meetings, the observer was positioned near, but not directly within, the physical space of the handover. Data were entered into the observational tool anonymously and stored securely on a password-protected computer for the duration of the study.

*Staff interviews, focus groups, and survey*

JR conducted unstructured field interviews with any available attendees (NCHDs) not engaged in patient care activities before or after the observed handover meetings. Questions were chosen to elaborate on findings from the observed meeting and to enquire about the attendee’s activities surrounding the handover process.

During phase II, to supplement final analysis and expand on quantitative findings, senior registrars representing all general surgical subspecialties in both sites were invited to take part in further interviews over an encrypted messaging application and participate in virtual focus groups.^14^ Phase I findings, including the differences in e-handover availability and dedicated handover location between the sites were presented during both interviews and focus groups to gather participant feedback on the differences observed. During focus groups, questions also evaluated participant perceptions of the surgical handover process and explored barriers and facilitators. As they were the most common handover leaders during observed meetings, senior registrars were chosen to take part in these interviews and focus groups to provide more detailed qualitative data.

Lastly, a cross-sectional survey was distributed to all surgical NCHDs working in both departments. It included the following open-ended questions: ‘What would you like to change about handover in your hospital? Do you have any suggestions for improvement?’.

**Data analysis**

*Real-time coding of verbal handover*

During the pilot observation period, details of full patient presentations were recorded by the observer. This was time-consuming and led to missed data. As an alternative, a list of handover-related codes (Table S3) was developed by the study team based on published handover methods^15,16^, and traditional medical school patient presentations^17^, allowing the observer to quickly and anonymously code handover content in real-time.

*Qualitative analysis*

Two authors (JR and AS) independently analysed pooled qualitative data from interviews, focus groups, and survey responses using inductive thematic analysis.^18^ They defined preliminary coding and met periodically to elaborate themes after coding completion. Software (NVivo qualitative data analysis software; QSR International Pty Ltd. Version 12.7.0, 2019) aided data management. The whole team reviewed these themes to refine the final analysis.

*Quantitative analysis*

Data were analysed using Stata (17.0©2021, StataCorp, Texas). Descriptive data are presented as absolute values and percentages, while continuous data are presented as mean (standard deviation) or median (range). Comparative analyses of quantitative data were performed using the chi-squared test for categorical variables to assess differences in distractions, interruptions, and electronic handover availability between sites. All tests of significance were two-tailed, with a significance level of p<0.05.

**DISCUSSION**

This study combined quantitative assessment of the handover process with rapid ethnography to compare institutional performance with international guidelines and enable qualitative exploration of contextual factors and behaviours. Unlike conventional ethnography which requires extended periods of observation,^19^ rapid ethnography uses trained observers^13^ and immediate engagement and collaboration with participants^12^ to hasten data collection. This approach is suited to healthcare contexts as it can be carried out in a short time-frame, thus minimising disruption to staff in complex clinical environments, and providing actionable findings in a timely fashion.^13^ Digital recordings are also problematic in the clinical context due to privacy concerns, and so a real-time coding system for verbal handover was developed to increase the efficiency and accuracy of field notes. The application of real-time coding has proven effective in the process evaluation of other team-based activities.^20,21^

Several studies have used audit to evaluate this process^22,23^; however, quantitative assessment of this complex team-based process yields limited information, and provides only superficial understanding of barriers and facilitators. While this study was not powered to detect differences between the sites, comparative analysis still enabled focused qualitative exploration of the findings. For example, only one hospital had a dedicated handover venue, and while no differences in noise-based interruptions or breaches of patient confidentiality were detected, participants clearly identified benefits in predictability of handover location and access to IT resources. One hospital was also noted to have significantly less available e-handovers. On questioning staff in the other hospital, they cited active consultant involvement and a strong handover culture as likely factors contributing to this difference. No previous interventional studies of daily surgical handover have rigorously applied an implementation framework to support their methodology which is a worthwhile focus for future studies. This approach requires an in-depth understanding of local context.^24^ Thus, beyond these local findings, this simple yet replicable methodology would enable clinicians in other settings to use this approach even in personnel and resource-constrained settings.

This study demonstrates that several guideline recommendations have not yet been implemented in the departments studied, with demonstrable impact on handover quality. For example, handover times should be standardised.^4-7^ In this study, consultants caused a large number of interruptions, but due to variable start times, were likely unaware handover was even taking place. Due to the clear patient safety risk of poor handover practices,^25,26^ hospital self-assessments should be carried out in all areas of handover to determine guideline compliance and identify areas for improvement. This methodology provides a rapid, cheap, and straightforward method to achieve these aims.

*Limitations*

All observations were carried out by a single observer, who was a surgical trainee. This may have shaped interpretations and preconceptions.^27^ However, this familiarity facilitated access and increased rapport, which are both necessary to carry out ethnography,^28^ and subject matter expertise was considered a strength of the study and safeguarded patient confidentiality. The study team was also comprised of researchers with varying backgrounds, including surgical consultants and a medical educationalist. This team developed a structured system to capture data and reduce subjectivity; however, future research may benefit from involvement of multiple observers from diverse backgrounds, to capture a broader view of handover practices. Participants were also aware they were being observed, which could have impacted behaviour.^29^ Recording of handover meetings was not used due to the data protection risks to patients and staff associated with storage and processing of recordings. Additionally, it would not be ethical to record or observe participants (staff) without their knowledge. International guidelines recommend multidisciplinary involvement,^5,6^ however, it was not routine practice for multidisciplinary providers to take part in the handover meetings at either location. Their perspectives were not evaluated during this study but future qualitative research assessing the impact of their inclusion in surgical handover would be beneficial. While this study provides rich insights into surgical handover practices in two urban academic teaching hospitals in Ireland, the findings may not be transferable to other institutions. However, while barriers to and enablers of best practice may vary in other settings, the mixed-methods assessment framework described here could enable similar assessments in other hospitals, even those with limited resources.

**Appendix S2. Handover observational tool**

| **Basics** | |
| --- | --- |
| Date |  |
| Type of handover |  |
| Relevant context |  |
| Did a handover meeting occur? (y/n) |  |
| If not, was there an opportunity to carry out a handover meeting? Please give further details |  |
| Handover method |  |
| Planned start time |  |
| Actual start |  |
| Reason for delay |  |
| End time |  |
| Location |  |
| Did planned location change and why? |  |
| **Team** | |
| Night Registrar on call for own consultant? |  |
| Night staff present |  |
| Day staff present |  |
| Handover leader & job title |  |
| Night staff permitted to depart after meeting? |  |
| **Equipment/tools** | |
| Were there working computers? |  |
| Were phones available? |  |
| Who printed the patient list? |  |
| Who printed the signout? |  |
| Who printed the patient bloods? |  |
| Were other tools used? |  |
| Patient locations checked pre-handover and by whom? |  |
| **Content** | |
| Free text |  |
| **Handover details:**  Opening statement  Coded handovers |  |
| Sick patients highlighted and discussed first? |  |
| Admissions/transfers |  |
| Consults |  |
| Floor issues |  |
| Discharges |  |
| Illness severities highlighted during individual presentations? |  |
| Action list |  |
| Actions assigned (patients) |  |
| Input from day team |  |
| Summary or readback encouraged |  |
| Operational matters |  |
| Summary statement |  |
| Prioritisation of tasks |  |
| Priority list for scans |  |
| Priority list for theatre |  |
| **Environment** | |
| Interruptions/distractions during handover |  |
| Other distractions |  |
| Description of physical space |  |

| **Handover timeline** | |
| --- | --- |
| Time | event |
|  |  |
|  |  |
|  |  |
|  |  |

| **Team interviews** | |
| --- | --- |
|  |  |
|  |  |

**Supplementary Figures and Tables**

| **Table S3. Handover observation codes** | | |
| --- | --- | --- |
| **Code** | **Short-hand code** | **Description** |
| Bottom line | BL | A brief, higher-order/executive summary:  e.g., ‘This 18 year-old patient likely has appendicitis and is for theatre today’,  ‘This 80 year-old woman has CT-confirmed uncomplicated diverticulitis and is for conservative management’ |
| Illness severity | IS | Any indication of how ‘well’ or ‘sick’ a patient is:  e.g., ‘This patient is critically unwell and for transfer to ICU’,  ‘This patient is stable at the moment but they need to be closely monitored today’ |
| Presenting complaint | PC | The reason the patient presented to hospital |
| Background | BG | Any details of the patient’s past medical or surgical history, current medications, allergies, social history, or family history |
| Assessment | Ax | Details of clinical examination, bedside, laboratory, or radiological investigations |
| Diagnosis | Dx | The working diagnosis for the patient’s condition |
| Differential diagnosis | Ddx | A list of potential diagnoses that have not been confirmed but which may have led to the patient’s condition |
| Progress |  | Any treatment administered to date or change in status of the patient |
| Plan |  | Investigations, treatment, etc, which are intended to be carried out after the handover |
| Contingency plan | Conting. | An evaluation/description of other eventualities/likely clinical scenarios  E.g., ‘If the scan shows appendicitis, the patient should undergo laparoscopic appendicectomy today’,  ‘This patient may deteriorate further and require transfer to ICU today’ |
| Educational point | Educ. | Any opportunity taken to educate junior members of staff during the handover |
| Question/comment from team | Q (insert rank) | Input from staff receiving the handover |
| Question to team | Q (leader) | Question from handover leader to other members of the team |
| Prioritisation | Priority | An indication of the order of importance of any tasks which require completion after the handover  E.g., scans, surgical cases, consults, etc |
| Action assigned | Action | Allocation of a task to a member of the team |
| Readback |  | Summary or readback by handover receiver |

| **Table S4. Summary of guideline recommendations used to guide observations^4-9^** | |
| --- | --- |
| **Recommendation category** | **Recommendations** |
| **Handover setup** | |
| ***Timing*** | Handover should be at a fixed time & punctuality is a key requirement |
|  | Bleep-free/protected time |
|  | There should be a handover at every change of shift (morning handover is the focus of study in this case) |
|  | Sufficient time should be set aside (it is expected to take about 30 minutes) |
| ***Attendance*** | Handovers should be multidisciplinary where appropriate with involvement of the nurse clinical coordinator/bed manager |
|  | All necessary grades of staff should attend |
| ***Leadership*** | There should be clear leadership, supervised by most senior clinician present |
|  | With daily senior clinician involvement |
| ***Location*** | It should be conducted verbally and face-to-face where possible (if not possible, consider phone or teleconference) |
|  | It should take place in a dedicated space, i.e., Not in use by others at the time |
|  | Close to the most used area of work |
|  | Large enough to allow everyone to attend |
|  | Free from distractions |
|  | In an environment which maintains patient confidentiality |
| ***Patient information resources & handover tools*** | *Computers:* Laboratory results, radiology system, intranet/internet, a system to identify inpatients |
|  | Clinical information/electronic records |
|  | Telephones |
|  | Supported with relevant, accurate and up-to-date documentation, including electronic media to support handover, with automation of data entry |
|  | Patient census sorted by location which identifies urgency of review |
|  | A handover checklist can be considered |
| **Handover content/structure** | |
| ***General structure/process*** | Leader to begin with a short introductory briefing to facilitate situational awareness |
|  | Team members should be alerted to clinically unstable patients at the outset |
|  | There should only be one speaker at a time |
|  | Two-way communication process – Questions/input from the team to be encouraged |
|  | Tasks should be prioritised |
|  | Operational issues should be discussed |
|  | The leader should promote read back of patient information, to verify receival |
| ***Verbal handover content*** | A handover method should be used – e.g., ISBAR, I-PASS, SIGNOUT |
|  | Information given should be succinct/relevant |
|  | Patient condition/illness severity should be clarified at the outset of each presentation |
|  | Current diagnosis |
|  | Relevant background |
|  | Results of significant or pending investigations |
|  | Anticipated issues/contingency plans |
|  | Management plans |
|  | Action list (and assignment of outstanding tasks) |

| **Table S5. Observations relating to the handover setup assessed using audit and ethnography** | | | |
| --- | --- | --- | --- |
|  | **Overall** | **Hospital A** | **Hospital B** |
| ***Timing/duration*** | | | |
| Occurrence of handover (n,%) | 26 (96.3) | 14 (100) | 12 (92.3) |
| Planned start time (median (range)) | 07:00 (06:15-09:00) | 06:52 (06:15-07:00) | 07:30 (07:00-09:00) |
| Start time (median (range)) | 07:05 (06:37-08:55) | 07:00 (06:37-07:14) | 07:29 (07:05-08:55) |
| Duration (minutes) median (range) | 12 (1-21) | 10 (1-21) | 12 (4-20) |
| Delay (minutes) median (range) | 11 (2-22) | 10 (1-22) | 10 (5-15) |
| Protected (bleep-free) time | No | No | No |
| ***Staff attendance*** | | | |
| Attendees (median, range) | 4 (2-7) | 4 (2-7) | 3 (2-4) |
| All grades in attendance (n,%) | 0 (0) | 0 (0) | 0 (0) |
| Multidisciplinary (n,%) | 0 (0) | 0 (0) | 0 (0) |
| Consultant present (n,%) | 3 (11.5) | 2 (15.4) | 1 (8.3) |
| ***Leadership (n,%)*** | | | |
| Clear leadership | 26 (100) | 14 (100) | 12 (100) |
| Most senior clinician present | 26 (100) | 14 (100) | 12 (100) |
| ***Location (n,%)*** | | | |
| Face-to-face | 26 (100) | 14 (100) | 12 (100) |
| Dedicated space (not shared) | 1 (3.8) | 1 (7.1) | 0 (0) |
| Extraneous noise | 9 (34.6) | 3 (21.4) | 6 (42.8) |
| Total number of interruptions  *Consultant*  *Another team*  *Communication/documentation*  *Own team*  *Call from ED/ward about a patient* | 17  7  4  3  1  2 | 7 (41.2)  2 (28.5)  1 (25)  3 (100)  1 (100)  0 (0) | 10 (58.8)  5 (71.4)  3 (75)  0 (0)  0 (0)  2 (100) |
| Interruptions (median (range)) | 0.5 (0-2) | 0 (0-2) | 1 (0-2) |
| ***Patient information resources (n,%)*** | | | |
| Working computers | 26 (100) | 14 (100) | 12 (100) |
| Printers | 24 (92.3) | 13 (92.8) | 11 (91.6) |
| Telephones | 26 (100) | 14 (100) | 12 (100) |
| Electronic handover | 15 (57.7) | 5 (35.7) | 10 (83.3) |
| Patient census | 22 (84.6) | 12 (85.7) | 10 (83.3) |
| Written handover | 6 (23.1) | 6 (42.8) | 0 (0) |

**Figure S6. Content and order of observed handovers**

**
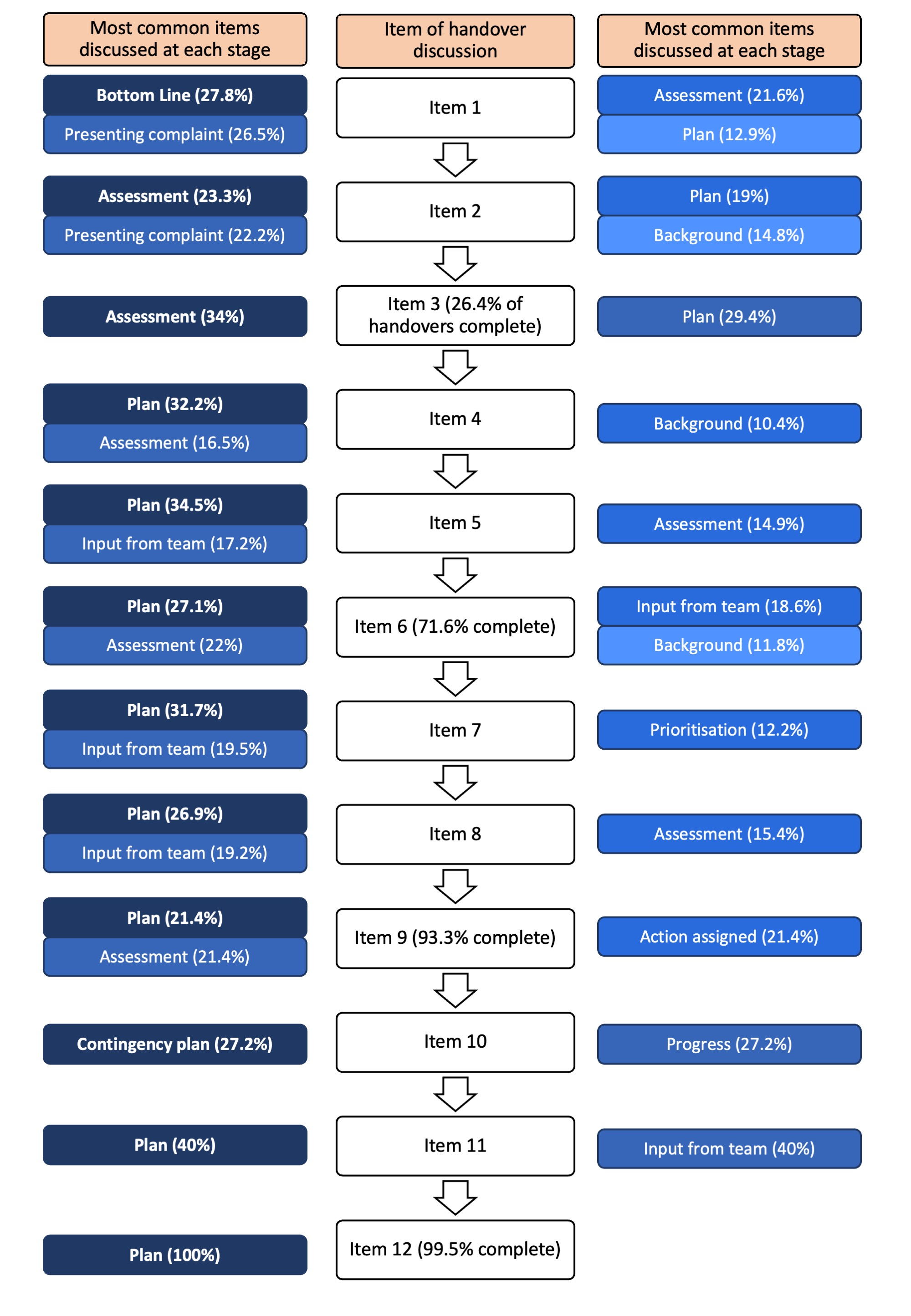
**

| **Table S7. Qualitative findings** | | |
| --- | --- | --- |
| **Theme** |  | **Example quotes** |
| **1** | **Significant invisible work surrounds the handover process** | |
|  | **Intern activities**   - - - Preparing and printing documents     - Checking patient locations | ‘Interns come in 15-20 mins early to do their pre-ward round jobs.’ *– Intern, field interview* |
|  | **SHO activities**   - Pre-handover ward rounds - Reviewing last minute referrals - Preparing e-handover - Printing e-handover | ‘I suppose what often happens is the [senior registrar] comes in… and often just gets the SHO to show them around to all of the patients…. so the SHO then ends up not getting to send it off before the rest of the team come in.’ *–SR, interview* |
|  | **Registrar activities**   - - - Pre-handover ward rounds     - Reviewing last minute referrals     - Ensuring e-handover is completed on time | ‘a flurry of last minute referrals… where you're not really sure whether you can stop accepting referrals because you're technically on until eight but consultants will do a round often at seven or whatever.’ *– SR, focus group* |
| **2** | **Factors influencing effective handover practice** | |
|  | **Barriers to handover** | |
|  | **Challenges with documentation**   - Different documents are in use - Documents can contain errors - Factors impacting documentation availability: - Competing clinical activities - Some staff are not given access to the e-handover - A lack of time | ‘…then you all of a sudden have three different documents, which is incredibly impractical.’  *– SR, focus group*  ‘It's largely inaccurate a lot of the time, it's rarely completely updated, often fields are left totally unpopulated... I don't really trust the information in it.’ *– SR, focus group*  ‘..interns do not receive surgical sign out which would be helpful.’ *– Survey response* |
|  | **Challenges with IT**   - - - Difficulty accessing computers     - Difficulty accessing printers     - Locum staff may not receive IT logins     - Staff can be unfamiliar with IT systems | ‘…you're trying to find space in the [ED], you're often at the mercy of whether the printers are working… you're battling for access to those computers with secretarial staff, medical staff, ED staff.’  *– SR, focus group*  ‘SHOs don’t know how to print [the e-handover] either even though it falls to them.’  *– SR, interview* |
|  | **The lack of a dedicated space for handover and the impact on:**   - IT access - Patient confidentiality - Distractions | ‘You need somewhere, just anywhere where you can sit down and be quiet and have a computer. No distractions from nurses, a computer to look at scans and bloods. That's the most frustrating thing.’  *– SR, focus group*  ‘you should be doing it in an area where patients can't overhear you and all this kind of stuff and we just don't have that.’ *– SR, focus group*  ‘Often handover in the middle of ED can be a disruptive environment. Handover meeting would be better in quiet room.’ *– Survey response* |
|  | **Consultant and junior staff attendance can be poor** | ‘but there are certain consultants who do come at the start of the round and they don't do handover and they're just like, "Bring me to the patient," and you're there and you're like, "Okay, well I need to tell you about the fact that she cries every time you ask her about this." And they're there like barrelling in and you're there trying to haul them back.’  *– SR, focus group*  ‘sometimes it is only that you meet the other SPR and the rest of the team don't arrive or they're off doing other things’ *– SR, focus group* |
|  | **Facilitators of handover** | |
|  | **Improving documentation**   - - - Increasing automation     - Using electronic patient records     - Improving the format of the e-handover | ‘They want the [patient] locations auto populated…’  *– Intern, field interview*  ‘A computer based program which will include all the data under single reporting… It will minimise any risk.’ *– Survey response*  ‘..the front summary could actually be so much more effective, have much more useful information.’  *– SR, focus group* |
|  | **Standardisation of the handover process**   - Providing protected handover time - Standardising the handover time and place - Providing office space with IT access | ‘Mandatory cut off times for referrals from ED unless emergencies. For example the last hour prior to handover to facilitate having the signout document completed.’ – *Survey response*  ‘Everyone just knows that the handover will happen in the office at the time that’s been dictated.’  *– SR, interview*  ‘..in other places it's easier here in general to get it done quickly and on time because of, I suppose there's dedicated relatively big general surgery office that always usually has a computer free with a printer that actually works 95% of the time.’  *– SR, focus group* |
|  | **Consultant oversight** | ‘I think that's a huge part of why [the e-handover is] so well used here, because all the consultants are very involved with it… it was the fear of [consultant] texting you at seven o'clock in the morning that it hadn't been sent.’ *– SR, focus group* |
|  | **A strong handover culture** | ‘In my opinion it is about the culture…What I mean is that on every level from consultant to intern the handover is crucial.’ *– SR, interview* |

*SHO, Senior House Officer; SR, Senior Registrar; ED, Emergency Department; IT, information technology; SpR, Specialist Registrar

**References**

1. Creswell JW, Clark VLP, Gutmann ML, Hanson WE. ADVANCED MIXED. Handbook of mixed methods in social & behavioral research. 2003:209.

2. Cuschieri S. The STROBE guidelines. Saudi journal of anaesthesia. 2019;13(Suppl 1):S31.

3. O'cathain A, Murphy E, Nicholl J. The quality of mixed methods studies in health services research. Journal of health services research & policy. 2008;13(2):92-8.

4. England RCoSo. Safe handover: guidance from the working time directive working party. RCS London; 2007.

5. Bywaters E, Calvert S, Eccles S, Eunson G, Macklin D, McCullough C, et al. Safe handover: safe patients. 2004.

6. Association AM. Safe handover: safe patients. Guidance on clinical handover for clinicians and managers. Canberra: AMA, 2006.

7. Committee on Acute Care Surgery, Canadian Association of General Surgeons. Clinical Practice Guideline: Dynamic Practice Guidelines for Emergency General Surgery2018 March 20 2023. Available from: <https://cags-accg.ca/wp-content/uploads/2018/11/ACS-Handbook-CPG-Ch-1-Rounding-and-Handover.pdf>.

8. Committee NCE. Communication (Clinical Handover) in Acute and Children’s Hospital Services, National Clinical Guideline.

9. Abdellatif A, Bagian JP, Barajas ER, Cohen M, Cousins D, Denham CR, et al. Communication during patient hand-overs: patient safety solutions, volume 1, solution 3, May 2007. Joint Commission Journal on Quality and Patient Safety. 2007;33(7):439-42.

10. Starmer AJ, Spector ND, Srivastava R, West DC, Rosenbluth G, Allen AD, et al. Changes in medical errors after implementation of a handoff program. New England Journal of Medicine. 2014;371(19):1803-12.

11. Aktinson P, Hammersley M. Ethnography and participant observation. Strategies of Qualitative Inquiry Thousand Oaks: Sage. 1998:248-61.

12. Pink S, Morgan J. Short‐term ethnography: Intense routes to knowing. Symbolic interaction. 2013;36(3):351-61.

13. Vindrola-Padros C, Vindrola-Padros B. Quick and dirty? A systematic review of the use of rapid ethnographies in healthcare organisation and delivery. BMJ Quality & Safety. 2018;27(4):321-30.

14. Stalmeijer RE, McNaughton N, Van Mook WN. Using focus groups in medical education research: AMEE Guide No. 91. Med Teach. 2014;36(11):923-39.

15. Starmer AJ, Spector ND, Srivastava R, Allen AD, Landrigan CP, Sectish TC, group I-Ps. I-pass, a mnemonic to standardize verbal handoffs. Pediatrics. 2012;129(2):201-4.

16. Haig KM, Sutton S, Whittington J. SBAR: a shared mental model for improving communication between clinicians. The joint commission journal on quality and patient safety. 2006;32(3):167-75.

17. Kurtz S, Silverman J, Benson J, Draper J. Marrying content and process in clinical method teaching: enhancing the Calgary–Cambridge guides. Academic Medicine. 2003;78(8):802-9.

18. Braun V, Clarke V. Using thematic analysis in psychology. Qualitative research in psychology. 2006;3(2):77-101.

19. Hammersley M, Atkinson P. Ethnography: Principles in practice: Routledge; 2019.

20. Schraagen JM, Schouten T, Smit M, Haas F, van der Beek D, van de Ven J, Barach P. A prospective study of paediatric cardiac surgical microsystems: assessing the relationships between non-routine events, teamwork and patient outcomes. BMJ Qual Saf. 2011;20(7):599-603.

21. Schraagen JM, Schouten T, Smit M, Haas F, van der Beek D, van de Ven J, Barach P. Improving methods for assessing teamwork in paediatric cardiac surgery. Qual Saf Health Care. 2010;19:e29.

22. Bakti NI, Williamson M, Sehjal R, Thilagarajah M. The use of microsoft excel as an electronic database for handover and coordination of patients with trauma in a district general hospital. BMJ Innovations. 2017;3(3):130-6.

23. Blower EL, MacCarrick T, Forster H, Sutton PA, Vimalachandran D. Implementation of a new tool to improve the efficacy and safety of surgical handovers. Int J Surg. 2015;13:189-92.

24. Bauer MS, Damschroder L, Hagedorn H, Smith J, Kilbourne AM. An introduction to implementation science for the non-specialist. BMC psychology. 2015;3(1):1-12.

25. Bigham MT, Logsdon TR, Manicone PE, Landrigan CP, Hayes LW, Randall KH, et al. Decreasing handoff-related care failures in children's hospitals. Pediatrics. 2014;134(2):e572-9.

26. Horwitz LI, Moin T, Krumholz HM, Wang L, Bradley EH. Consequences of inadequate sign-out for patient care. Archives of Internal Medicine. 2008;168(16):1755-60.

27. Mahtani K, Spencer EA, Brassey J, Heneghan C. Catalogue of bias: observer bias. BMJ evidence-based medicine. 2018;23(1):23-4.

28. Morgan-Trimmer S, Wood F. Ethnographic methods for process evaluations of complex health behaviour interventions. Trials. 2016;17(1):232.

29. Demetriou C, Hu L, Smith TO, Hing CB. Hawthorne effect on surgical studies. ANZ Journal of Surgery. 2019;89(12):1567-76.
